# Contemporary organosilicon chemistry

**DOI:** 10.1186/1860-5397-3-4

**Published:** 2007-02-08

**Authors:** Steve Marsden

**Affiliations:** 1 School of Chemistry, University of Leeds, Leeds LS2 9JT, UK

## Abstract

Editorial for the Thematic Series on Contemporary Organosilicon Chemistry.

The field of organosilicon chemistry has a rich and varied history, and has long since made the progression from chemical esoterica to its position as a mainstay of modern synthetic chemistry. In his 1980 Tilden lectures [1], Professor Ian Fleming of Cambridge University, himself one of the major practitioners of the discipline, identified the year 1968 as a watershed in the popularisation of organosilicon chemistry. Notwithstanding the earlier, pioneering work of chemists such as Eaborn, 1968 was notable for many innovations we now take for granted, including the development of silyl enol ether chemistry by Stork and Hudrlik, and the eponymous olefination reaction by Peterson. These landmark papers triggered a massive growth in interest in the area which continues to this day.

Nearly 40 years on from those landmark publications, one could be forgiven for assuming that organosilicon chemistry has reached such a state of maturity that there remain few areas ripe for new development. A brief survey of the modern literature quickly dispels this notion. Far from atrophying, organosilicon chemistry continues to be an area of expansion, with an average of over 550 papers being published per year in the decade to date – an increase of over 30% by comparison with the 1990s, and equivalent to the number appearing in the much longer established field of organoboron chemistry [2]. This expansion in activity reflects not only the sustained popularity of traditional silicon-based reactions and reagents, but also newer departures such as the effective application of organosilicon compounds in transition metal-catalysed cross-coupling reactions, and the use of silanes as stoichiometric reductants in a range of chemo-, stereo- and enantioselective catalytic reductions.

It is therefore a pleasure to serve as Guest Editor for this first "Thematic Series" in the Beilstein Journal of Organic Chemistry, on "Contemporary Organosilicon Chemistry". We have contributions from some of the leading practitioners in the area, covering a wide range of topics including the stereoselective construction of oxygen and nitrogen-containing heterocycles, the use of tethered silicon reagents to deliver acyclic stereocontrol, chiral-at-silicon reagents for asymmetric synthesis, and a new method for the electrochemical generation of silyl cations. Additionally, the unique nature of internet-based publishing means that the Series can grow as additional contributions are received: future papers in areas including allylation chemistry, stereoselective fluorination, cyclopropane chemistry and the development of silicon-containing drug candidates should be available shortly. Be sure to check back to keep abreast of the latest developments as the Series grows.

Steve Marsden

Guest Editor
